# Age and Sex Specific Reference Intervals for Modifiable Risk Factors of Cardiovascular Diseases for Gujarati Asian Indians

**DOI:** 10.1155/2015/394798

**Published:** 2015-12-28

**Authors:** Sibasis Sahoo, Komal H. Shah, Ashwati R. Konat, Kamal H. Sharma, Payal Tripathi

**Affiliations:** ^1^Department of Cardiology, U. N. Mehta Institute of Cardiology and Research Centre (UNMICRC), Asarwa, Ahmedabad 380016, India; ^2^Research Department, U. N. Mehta Institute of Cardiology and Research Centre (UNMICRC), Asarwa, Ahmedabad 380016, India; ^3^Pathology Department, U. N. Mehta Institute of Cardiology and Research Centre (UNMICRC), Asarwa, Ahmedabad 380016, India

## Abstract

*Objective*. We aimed to establish age and sex specific percentile reference data for cardiovascular risk factors such as lipids, sugar, blood pressure, and BMI in apparently healthy and disease-free Gujarati population.* Methods.* In this cross-sectional study, we enrolled 3265 apparently healthy and disease-free individuals of both genders residing in Gujarat state. Fasting samples of blood were used for biochemical estimations of lipids and sugar. The measurement of BMI and blood pressure was also done according to the standard guidelines. Age and gender specific 5th, 25th, 50th, 75th, 90th, and 95th percentiles were obtained.* Results*. The mean values of lipids, sugar, blood pressure, and BMI were significantly (*p* < 0.001) higher in males as compared to female population. Age-wise distribution trends showed increase in the risk factors from the 2nd decade until the 5th to 6th decade in most of the cases, where loss of premenopausal protection in females was also observed. Specific trends according to gender and age were observed in percentile values of various parameters.* Conclusion.* The outcome of current study will contribute significantly to proposing clinically important reference values of various lipids, sugar, blood pressure, and BMI that could be used to screen the asymptomatic Gujarati Indian population with a propensity of developing dyslipidemia, diabetes, blood pressure, and obesity.

## 1. Introduction

South Asians, especially Indians, are group of communities bearing genetically diverse ethnic characteristics and are passing through epidemic health shift due to urbanization [1]. This leads to a marked increase in the incidence of noncommunicable diseases such as cardiovascular diseases (CVDs), which are subjected to race-ethnic diversity and are longitudinally related to the classical risk profile of the particular community [2]. Mortality and morbidity statistics of the last few decades had reported substantial increase and regional variation in cardiovascular epidemic trends in India [3]. Hence, it is noteworthy that despite similar risk factor profile the prediction and interpretation of mortality and morbidity in Indians are prone to ethnic and geographic variations. Socioeconomic status and presence of modifiable risk factors such as individual life style factors, lipid levels, diabetes, and hypertension are noted as the proximate/primordial determinants of CVDs [4].

The circulating lipid levels provide substantially good evidence of progressive atherosclerosis in asymptomatic individuals [5, 6]. Reports from various individual studies have documented steep rise in mean values of Total Cholesterol (TC), Low Density Lipoprotein Cholesterol (LDL-C) and non-High Density Lipoprotein Cholesterol (non-HDL-C), and Triglyceride (TG) and fall in HDL-C in Indians [7]. A population based survey had reported a great degree of variation in lipid associated allele frequencies across multiple racial and ethnic population [8]. Moreover, the evaluation of ethnicity based factors on lipid levels in various communities was recommended by Third Report of the National Cholesterol Education Program Expert Panel on Detection, Evaluation, and Treatment of High Blood Cholesterol in Adults (NCEP-ATP III) also [9]. Hence, expert panels from various regions of world have established well-defined guidelines for reference values of lipids, and following this in India also various states have proposed the reference ranges for lipids exclusive for particular geographic and ethnic population [10–12]. In the same manner, racial disparities in diabetes, hypertension, and obesity are reported by many.

Gujarat is a rich state located in the western region of India. Numerous studies conducted on native and migrated Gujarati populations have reported that this community possesses different cardiovascular risk factor profile as compared to other ethnic groups [13, 14]. Apart from the fact that it has the highest number of newly diagnosed diabetic population, it also has considerably high incidence of smokeless tobacco consumption, obesity, and dyslipidemias [15–17]. The earlier attempt of comparing the mean values of lipid profile components of healthy Gujaratis with other Indian communities showed a great degree of variations between these groups [18]. Moreover, the age and sex specific representative percentile reference data for lipids, sugar, hypertension, and Body Mass Index (BMI) of Gujarati population is lacking. Keeping in mind the lacuna of research, current study was designed to establish the percentile values for serum lipids and sugar, blood pressure, and BMI in healthy Gujarati male and female in order to provide reference data of clinical relevance.

## 2. Methods

### 2.1. Design and Data Collection

This cross-sectional study was conducted in September 2013 at U. N. Mehta Institute of Cardiology and Research Center which was approved and cleared by institutional ethics committee. In the current investigation, 3265 individuals of both genders, who were apparently healthy, free from any illness and major complications or surgery for the last three months with normal BP, ECG, ECHO, and stress test, were selected. The sample was taken from the self-reported healthy population who visited the hospital during awareness camp. The population was from various regions of Gujarat state. The demographic details of the population such as age, sex, and disease history were recorded for all the individuals. The subjects were asked to relax and then the blood pressure was measured for three times and the average was taken into account. All clinical investigations were conducted according to the principles expressed in the Declaration of Helsinki.

### 2.2. Laboratory Tests

Fasting serum lipids and glucose concentrations were measured by International Federation of Clinical Chemistry (IFCC) approved enzymatic methods using commercially available kit on autoanalyzer (ARCHITECT PLUS ci4100, Germany). Lipids levels were classified according to the National Cholesterol Education Program (NCEP) and Adult Treatment Panel III (ATP III) guidelines. Blood pressure of the population was measured according to earlier reported guidelines [19]. Individuals having BMI greater than 30 were considered to be obese as per WHO standards [20].

### 2.3. Quality Control

The accuracy and precision of laboratory parameters were achieved by participating in the external and internal quality control programs of CMC, Vellore, and Bio-Rad. To maintain the reproducibility and reliability of the test, the quality assessment programs were routinely practiced and accordingly the values were standardized for mean ± 1 standard deviation (SD). For internal quality control, the accuracy of reports was tested by retesting the same sample within the hospital. Performance of all results was done following quality control program based on west grade QC rule like 1-2s as warning rule and 2-2s, 1-3s, and 1-4s as rejection rule. Test performance was started with acceptable QC range. In case of rejection (QC outlier), recalibration and use of fresh control analysis were done. For external quality control, the sample was sent to reputed NABL accredited laboratories for interlab comparison and verification of accuracy of the reports. In addition, the participation in periodic EQAS Programs for Haematology, Clinical Chemistry and Immunoassay was done on the basis of AIIMS, Delhi, Bio-Rad, USA, respectively. The data obtained from everyday quality control check was expressed in terms of SD and Coefficient of Variation (CV) and is presented in Table 1.

### 2.4. Statistical Analysis

All collected data were analyzed by SPSS v. 20 (Chicago, IL, USA). Distribution analysis showed that most of the parameters follow non-Gaussian distribution. Mean, median, and percentile data of 5th, 25th, 50th, 75th, and 95th were calculated for the values of lipids, glucose, blood pressure, and BMI.

## 3. Results

The overall results obtained from the current investigation are presented in Table 2 as mean ± SD and median of lipid profile, glucose, blood pressure, and BMI in Gujarati Indian population.

Our results showed mean ± SD of cholesterol to be 185.33 ± 39.24 mg/dL and Triglyceride in the studied population was found to be 115.59 ± 63.47 mg/dL. The mean ± SD was 118.5 ± 33.45 mg/dL for LDL-C and 43.7 ± 9.87 mg/dL for HDL-C and 23.11 ± 12.69 mg/dL for VLDL. The mean reference values obtained for TL, LDL-C/HDL-C, and TC/HDL-C were 644.62 ± 88.02, 2.84 ± 1.02, and 4.42 ± 1.4 mg/dL, respectively. In the current study cohort females showed superior lipid profile (TC: 181.55 ± 38.93 versus 188.48 ± 39.2; TG: 100.63 ± 48.18 versus 127.91 ± 71.44; HDL-C: 47.52 ± 9.68 versus 40.56 ± 8.81; LDL-C: 113.89 ± 33.54 versus 122.34 ± 32.89; VLDL: 20.12 ± 9.63 versus 25.58 ± 14.28; TL: 629.72 ± 75.45 versus 656.96 ± 95.46; LDL-C/HDL-C: 2.49 ± 0.88 versus 3.12 ± 1.01; TC/HDL-C: 3.94 ± 1.07 versus 4.81 ± 1.46), glucose (83.08 ± 21.49 versus 88.64 ± 25.12), blood pressure (SBP: 126.47 ± 18.87 versus 133.74 ± 17.97; DBP: 80.67 ± 10.37 versus 84.14 ± 10.55), and BMI (26.61 ± 85.88 versus 27.09 ± 44.67) as compared to males, which was highly significant (*p* < 0.001; Table 2).

The percentile limits from 5th to 95th for various lipids, glucose, and blood pressure according to gender tabulated in Table 3 show higher cutoff values of all the variables in males as compared to females except for slight elevation in BMI levels in higher percentile values. Age-wise distribution trends analysis showed steep increase in the levels of lipids, glucose, blood pressure, and obesity from the 2nd decade till the 5th to 6th decade in most of the cases (Table 4), indicating natural ageing itself as one of the risk factors. In contrast to others, the level of HDL-C was found to increase with the advancing of age. The 5th to the 95th percentile values of lipids, glucose, SBP, DBP, and BMI in various age groups were also analyzed (Table 5). Most of the variables achieved stable levels after the 5th decade and in some cases a decline was observed after the 6th decade of life.

Comparative examination of reference values established for various communities based on the population mean has been presented in Table 6. We have observed that the TC of Gujarati population had the third highest (185.33 ± 39.245 mg/dL) mean value in comparison to other Indian populations, whereas LDL-C had the third highest (118.5 ± 33.46) mean value amongst all including Venezuelan and Nepali populations. The level of good cholesterol, HDL-C, was lowest (43.70 ± 9.870) in Gujarati population.

## 4. Discussion

The need for establishment of different reference intervals of lipids, sugar, blood pressure, and obesity in various community could be justified by the fact that these risk factors are subjected to racial and geographic variations with life style changes, demographic shifts, epidemiological transition, and urbanization playing a prime role. Moreover, Indians possess some of the unique characteristics such as higher predisposition to the development of type 2 diabetes mellitus, excess visceral fat and centripetal obesity, and smaller body size, which needs to be considered before screening the patients with any guidelines formerly standardized on western population [21]. Along with the “genetically deformed life style,” other modifiable risk factors of CVD such as dyslipidemia, diabetes, hypertension, and obesity are increasing in Gujarati population [22, 23]. A physically inactive life style and diet rich in oil and sugar have made Gujarat the second largest contributor of Indian diabetic population. This ethnic group is highly prone to develop and suffer from premature vascular ageing as their heart age is 6.7 years higher than their chronological age with dyslipidemia, hypertension, increased waist circumference, and diabetes as the involved factors [17]. Hence, it is of utmost importance to standardize the reference range of CVD contributing risk factor in Gujaratis. The first attempt to establish normal percentile values of serum lipid levels in healthy, vegetarian Gujarati population was done by Jhala et al. in 1998 [18]. The study showed natural ageing and gender specific disturbance in lipid profile. The author had reappraised the lipid values of the same community in 2009 [24] and compared the data with other populations from different states of India showing variation in lipid profile of Gujaratis as compared to others. In spite of the significant contribution of both studies in lipid reference range establishment, the findings lacked percentile values specific to various age groups and gender. Moreover, none of the studies has established reference range for blood glucose, blood pressure, and BMI in healthy, adult Gujarati population.

To the best of our knowledge, this is the first ever large study providing representative age and gender specific percentile values for serum lipids, glucose, blood pressure, and BMI in healthy Gujarati Indians. Contrary to earlier reports where no substantial sex specific difference was noted in the lipid profile [11, 25], we had obtained significant difference between the risk profiles of males and females of the study population. The females were having superior CVD risk factor profile due to the estrogenic protective effect, which was found to be lost in the 5th to the 6th decade of life.

Natural ageing induced elevation in CVD risk factors was observed in the study population; however, surprisingly, the mean value of HDL was found to increase steadily after the 3rd decade with the increasing age in both genders which is in accordance with other studies (Andhra Pradesh). A significant finding reflected from decade-wise analysis is the loss of premenopausal protection in females in the 5th to 6th decade of life as the mean values of serum Total Cholesterol, LDL-C, SBP, and BMI were higher in females of 50–59 age group as compared to their male counterpart of the same age group [11, 12]. Several epidemiological studies have shown the interplay between multiple risk factors of CVD such as dyslipidemia, diabetes, BMI, and hypertension [26, 27]. In concordance with our study, the coexistence of these risk factors in Gujarati population has been reported by others also [28]. One of the most striking outcomes of the study is the relatively low level of HDL-C in Gujaratis compared to Rajasthani, Andhra Pradesh, Bengali, Maharashtrian, Venezuelan, and Nepalese population [10, 12, 29–32]. All of these studies were undertaken on apparently healthy population similar to ours. Prajapati et al. had shown great degree of association between higher carotid intima thickness and low HDL-C [33] levels in Gujarati children, making HDL-C one of the strongest predictors of CAD in the studied community. We have observed the alarmingly high proportion of population being unaware of the CVD risk factors prevalence.

Representative percentile reference data of modifiable risk factors of CVD are highly required for the framing of appropriate diagnostic and preventive policies. The values provided in the current study could be effectively used as reference values that are specific for Gujarati population. The strengths of the study are the well-defined percentile values of modifiable risk factors, larger population size, standard laboratory techniques, and participants from different regions of Gujarat state. However, the study also possesses some limitations such as lack of information regarding the history of genetics, diet, and stress, daily consumption of alcohol and tobacco of the study population.

## 5. Conclusion

The outcome of the current study will contribute significantly to standardization of the reference values of various lipids, glucose, blood pressure, and BMI which are of clinical importance and will help in the designing of better diagnostic strategies.

## Figures and Tables

**Table 1:** Comparison of quality control material at levels I and II from Bio-Rad with the mean and percentage of Coefficient of Variations obtained from laboratory.

Analyte	Reference mean		Mean obtained		% of CV	
	Level I	Level II	Level I	Level II	Level I	Level II
TC (mg/dL)	249.6	104.4	250.1	103.9	3.4	0.92
TG (mg/dL)	182.5	95.9	182.3	95.2	1.4	1.06
HDL-C (mg/dL)	65.4	26.9	65.2	26.5	2.5	1.96
Blood sugar (mg/dL)	297.9	81.8	297.6	81.5	3.3	3.3

Level I: Bio-Rad control (abnormal); level II: Bio-Rad control (normal); CV: Coefficient of Variation; TC: Total Cholesterol; TG: Triglyceride; HDL-C: High Density Lipoprotein Cholesterol.

**Table 2:** Mean and median values of lipid profile, sugar, blood pressure, and BMI in Gujarati population.

Variable	Total	Male	Female
TC (mg/dL)			
Mean ± SD	185.33 ± 39.24	188.48 ± 39.20	181.55 ± 38.93
Median	182	187	178
TG (mg/dL)			
Mean ± SD	115.59 ± 63.47	127.91 ± 71.44	100.63 ± 48.18
Median	99	110	88
HDL-C (mg/dL)			
Mean ± SD	43.7 ± 9.87	40.56 ± 8.81	47.52 ± 9.68
Median	43	40	47
LDL-C (mg/dL)			
Mean ± SD	118.5 ± 33.45	122.34 ± 32.89	113.89 ± 33.54
Median	116	121.2	110
LDL-C/HDL-C			
Mean ± SD	2.84 ± 1.02	3.12 ± 1.01	2.49 ± 0.88
Median	2.71	3.05	2.37
TC/HDL-C			
Mean ± SD	4.42 ± 1.40	4.81 ± 1.46	3.94 ± 1.07
Median	4.26	4.68	3.77
VLDL (mg/dL)			
Mean ± SD	23.11 ± 12.69	25.58 ± 14.28	20.12 ± 9.63
Median	19.8	22	17.6
TL (mg/dL)			
Mean ± SD	644.62 ± 88.02	656.96 ± 95.46	629.72 ± 75.45
Median	629	641.5	617
Blood sugar (mg/dL)			
Mean ± SD	86.13 ± 23.71	88.64 ± 25.12	83.08 ± 21.49
Median	83	85	81
SBP (mm/Hg)			
Mean ± SD	130.45 ± 18.73	133.74 ± 17.97	126.47 ± 18.87
Median	130	130	122
DBP (mm/Hg)			
Mean ± SD	82.57 ± 10.61	84.14 ± 10.55	80.67 ± 10.37
Median	80	82	80
BMI (kg/m^2^)			
Mean ± SD	26.87 ± 66.52	27.09 ± 44.67	26.61 ± 85.88
Median	24.12	24.16	24.05

TC: Total Cholesterol; TG: Triglycerides; LDL-C: Low Density Lipoprotein Cholesterol; HDL-C: High Density Lipoprotein Cholesterol; VLDL: Very Low Density Lipoprotein; TL: Total Lipid; SBP: Systolic Blood Pressure; DBP: Diastolic Blood Pressure; BMI: Body Mass Index.

Data was expressed as mean ± SD. Significance was obtained between males and females using Mann-Whitney *U* Test. Level of significance was accepted at *p* < 0.05 level.

**Table 3:** Percentile values of lipid profile, sugar, blood pressure, and BMI in population.

		*n*	5th percentile	25th percentile	50th percentile	75th percentile	95th percentile	97th percentile
TC (mg/dL)	Men	1789	128	161	187	213.5	255	268.3
	Women	1476	127	154.25	178	204	250	262

TG (mg/dL)	Men	1789	59	83	110	152	257.5	293.5
	Women	1476	50.85	68	88	119	194.3	214

HDL-C (mg/dL)	Men	1789	29	35	40	45	55	58.3
	Women	1476	33	41	47	53	65	67

LDL-C (mg/dL)	Men	1789	71	99	121	142	178	188.3
	Women	1476	66.85	89	110	134	174	183

LDL-C/HDL-C	Men	1789	1.64	2.4	3.05	3.76	4.8	5.04
	Women	1476	1.25	1.83	2.37	3.02	4.08	4.39

TC/HDL-C	Men	1789	2.97	3.87	4.68	5.58	7.05	7.47
	Women	1476	2.5	3.16	3.77	4.6	5.91	6.26

VLDL (mg/dL)	Men	1789	12	17	22	30	51.5	58.3
	Women	1476	10	14	18	24	39	43

TL (mg/dL)	Men	1789	540	592	641	701	830	865
	Women	1476	531	577	617	672	772.15	799.69

Blood sugar (mg/dL)	Men	1789	65	76	85	94	120	139
	Women	1476	62	73	81	89	108	115

SBP (mm/Hg)	Men	1789	110	120	130	142	168	176
	Women	1476	100	112	122	136	162	176

DBP (mm/Hg)	Men	1789	70	80	82	90	100	100.6
	Women	1476	70	70	80	88	100	100

BMI (kg/m^2^)	Men	1789	17	22	24	27	32	34
	Women	1476	17	20	24	27	33	35

TC: Total Cholesterol; TG: Triglycerides; HDL-C: High Density Lipoprotein Cholesterol; LDL-C: Low Density Lipoprotein Cholesterol; VLDL: Very Low Density Lipoprotein; TL: Total Lipid; SBP: Systolic Blood Pressure; DBP: Diastolic Blood Pressure; BMI: Body Mass Index. Data are expressed as the 5th to 97th percentile of median.

**Table 4:** Decade-wise percentile values of lipid profile, sugar, blood pressure, and BMI in Gujarati population.

		20–29	30–39	40–49	50–59	60–69	>70
TC (mg/dL)	Total	161.29 ± 30.64	181.18 ± 178	193.10 ± 37.01	201.40 ± 38.81	201.31 ± 41.98	197.98 ± 37.94
	Male	165.93 ± 32.64	187.38 ± 37.99	193.43 ± 38.89	197.94 ± 38.69	195.83 ± 40.28	193.91 ± 33.42
	Female	158.15 ± 28.83	172.89 ± 30.15	192.70 ± 34.64	206.90 ± 38.51	213.04 ± 43.49	219.22 ± 53.58

TG (mg/dL)	Total	89.7 ± 42.60	116.05 ± 71.59	122.85 ± 66.11	131.56 ± 65.81	127.38 ± 58.36	128.28 ± 60.26
	Male	106.09 ± 50.87	131.09 ± 82.10	133.23 ± 76.71	138.90 ± 73.11	124.34 ± 40.28	124.65 ± 61.63
	Female	78.63 ± 31.50	95.95 ± 47.68	110.30 ± 47.54	119.87 ± 50.27	134.21 ± 62.95	147.22 ± 51.47

HDL-C (mg/dL)	Total	45.43 ± 10.56	42.18 ± 8.72	43.37 ± 9.34	43.44 ± 9.24	43.93 ± 11.93	44.12 ± 10.39
	Male	40.30 ± 8.22	39.56 ± 7.62	40.15 ± 7.89	40.87 ± 8.49	42.58 ± 12.88	43.08 ± 9.91
	Female	48.98 ± 10.57	45.69 ± 8.89	47.26 ± 9.48	47.51 ± 8.95	46.97 ± 8.75	49.55 ± 11.72

LDL-C (mg/dL)	Total	97.91 ± 26.15	115.78 ± 29.61	125.16 ± 31.80	131.64 ± 33.89	131.72 ± 35.81	128.2 ± 30.16
	Male	104.41 ± 27.69	121.59 ± 31.38	126.63 ± 32.55	129.28 ± 33.62	128.38 ± 33.81	125.89 ± 26.86
	Female	93.52 ± 24.12	108.01 ± 25.09	123.37 ± 30.83	135.41 ± 34.13	139.22 ± 39.11	140.22 ± 43.72

LDL-C/HDL-C	Total	2.28 ± 0.87	2.85 ± 2.71	3.00 ± 0.94	3.15 ± 1.06	3.13 ± 3.04	3.0 ± 0.82
	Male	2.70 ± 0.90	3.16 ± 0.95	3.24 ± 0.95	3.28 ± 1.13	3.17 ± 1.03	3.04 ± 0.87
	Female	2.00 ± 0.74	2.44 ± 0.70	2.70 ± 0.84	2.94 ± 0.91	3.04 ± 0.94	2.82 ± 0.53

TC/HDL-C	Total	3.72 ± 1.08	4.45 ± 1.19	4.62 ± 1.20	4.84 ± 1.77	4.76 ± 1.24	4.64 ± 1.11
	Male	4.26 ± 1.12	4.87 ± 1.22	4.95 ± 1.22	5.06 ± 2.06	4.81 ± 1.29	4.68 ± 1.19
	Female	3.35 ± 0.88	3.89 ± 0.88	4.21 ± 1.02	4.48 ± 1.08	4.65 ± 1.12	4.44 ± 0.60

VLDL (mg/dL)	Total	17.94 ± 8.52	23.21 ± 14.31	24.571 ± 13.22	26.31 ± 13.16	25.74 ± 11.67	25.65 ± 12.05
	Male	21.21 ± 10.17	26.21 ± 16.42	26.64 ± 15.34	27.78 ± 14.62	24.86 ± 11.21	24.93 ± 12.32
	Female	15.72 ± 6.30	19.19 ± 9.53	22.06 ± 9.50	23.97 ± 10.05	26.84 ± 12.59	29.44 ± 10.29

TL (mg/dL)	Total	596.42 ± 63.56	639.4 ± 93.55	659.33 ± 86.84	676.40 ± 85.69	672.44 ± 85.32	670.39 ± 82.16
	Male	612.33 ± 72.96	658.04 ± 105.79	666.82 ± 99.99	677.72 ± 92.57	662.75 ± 82.91	661.65 ± 79.75
	Female	585.68 ± 53.84	614.5 ± 66.55	650.26 ± 66.62	674.30 ± 73.77	694.23 ± 87.09	716.00 ± 83.97

Blood sugar (mg/dL)	Total	76.93 ± 11.06	82.80 ± 15.86	88.816 ± 23.10	93.49 ± 30.84	94.55 ± 36.14	89.39 ± 16.43
	Male	79.23 ± 11.02	85.16 ± 17.77	87.00 ± 17.68	95.61 ± 33.78	92.48 ± 26.06	90.85 ± 17.15
	Female	75.37 ± 10.81	79.64 ± 12.20	87.00 ± 17.68	90.12 ± 25.27	99.21 ± 52.04	81.77 ± 9.29

SBP (mm/Hg)	Total	120.5 ± 14.72	127.14 ± 16.49	133.28 ± 18.13	137.85 ± 19.06	139.53 ± 36.14	138.25 ± 16.36
	Male	128.23 ± 15.42	129.95 ± 15.85	134.44 ± 18.36	137.59 ± 18.35	139.76 ± 20.43	137.31 ± 15.28
	Female	115.28 ± 11.62	123.37 ± 16.60	131.87 ± 17.77	138.27 ± 20.20	139.01 ± 20.91	143.11 ± 21.61

DBP (mm/Hg)	Total	77.40 ± 9.04	81.70 ± 9.90	84.30 ± 9.92	86.25 ± 11.07	85.62 ± 11.66	83.28 ± 8.75
	Male	80.77 ± 8.46	83.02 ± 10.02	84.90 ± 9.83	86.31 ± 11.88	85.98 ± 12.13	83.06 ± 8.95
	Female	75.12 ± 8.71	79.94 ± 9.47	83.58 ± 9.99	86.15 ± 9.68	84.80 ± 10.54	84.44 ± 7.98

BMI (kg/m^2^)	Total	24.78 ± 42.60	24.40 ± 5.00	27.52 ± 44.00	30.98 ± 131.38	25.42 ± 6.32	36.99 ± 94.84
	Male	29.48 ± 66.63	24.33 ± 4.64	28.94 ± 59.24	25.23 ± 4.46	24.81 ± 4.20	39.40 ± 103.51
	Female	21.60 ± 4.55	24.50 ± 5.46	25.82 ± 5.70	40.12 ± 211.5	26.79 ± 9.39	24.39 ± 4.70

TC: Total Cholesterol; TG: Triglycerides; HDL-C: High Density Lipoprotein Cholesterol; LDL-C: Low Density Lipoprotein Cholesterol; VLDL: Very Low Density Lipoprotein; TL: Total Lipid; SBP: Systolic Blood Pressure; DBP: Diastolic Blood Pressure; BMI: Body Mass Index.

Data are expressed as mean ± SD.

**Table 5:** Decade-wise percentile values of lipid profile, sugar, blood pressure, and BMI in Gujarati population.

	Age in years	*n*	5th percentile	25th percentile	50th percentile	75th percentile	95th percentile	97th percentile
TC (mg/dL)	20–29	796	118	140.25	157	179.75	216	228.09
	30–39	653	128	157.5	178	201	242.3	254.38
	40–49	844	138	169	190	213	256.5	276.3
	50–59	627	140	175	200	226	270	278
	60–69	289	132.5	174	198	228.5	274	288.5
	>70	56	127.85	173.5	202.5	220.25	254.3	275.43

TG (mg/dL)	20–29	796	47.85	63	77	102.75	178.15	202.09
	30–39	653	52	72	98	141	230.3	278
	40–49	844	62	83	106	142.75	231.75	264.3
	50–59	627	63.4	88	115	153	271	292.8
	60–69	289	62.5	89	112	154	241.5	274.5
	>70	56	59	74.75	116.5	172.75	240.1	284.66

HDL-C (mg/dL)	20–29	796	30	38	44	52	65	67.09
	30–39	653	29	36	42	47	58	60
	40–49	844	31	37	42	49	61	63
	50–59	627	30	38	42	49	61	63
	60–69	289	30	37	42	49.5	60	63.3
	>70	56	30.85	36.25	41.5	51.25	64.15	66.74

LDL-C (mg/dL)	20–29	796	60	81	93	113	146	158
	30–39	653	71	94	114	133	169	178.38
	40–49	844	77.25	104	123	143	179	185
	50–59	627	77	109	131	153	185.6	200
	60–69	289	72.5	107.5	131	155	195	203
	>70	56	80.25	111	127	146.75	177.35	196.6

LDL-C/HDL-C	20–29	796	1.184	1.645	2.109	2.747	3.979	4.311
	30–39	653	1.573	2.185	2.717	3.414	4.498	4.792
	40–49	844	1.626	2.327	2.89	3.603	4.63	4.976
	50–59	627	1.719	2.476	3.045	3.711	4.871	5.249
	60–69	289	1.707	2.316	3.047	3.882	4.956	5.037
	>70	56	1.692	2.399	2.975	3.429	4.686	4.913

TC/HDL-C	20–29	796	2.359	2.924	3.474	4.31	5.74	6.195
	30–39	653	2.842	3.562	4.315	5.164	6.78	7.395
	40–49	844	2.935	3.729	4.477	5.353	6.61	7.276
	50–59	627	3.116	3.914	4.666	5.468	7	7.302
	60–69	289	3.008	3.805	4.738	5.602	6.88	7.278
	>70	56	2.974	3.868	4.361	5.373	6.91	7.52

VLDL (mg/dL)	20–29	796	9.85	13	15	20.75	36	40.09
	30–39	653	10	14.5	20	28	46	55.76
	40–49	844	12	17	21	28.75	46	53
	50–59	627	13	18	23	31	54.6	58.16
	60–69	289	12.5	18	22	31	48	55.3
	>70	56	12	15	23.5	34.75	48.05	57.19

TL (mg/dL)	20–29	796	520	555	585	623	722	763.09
	30–39	653	533	579	623	677	791.9	849.9
	40–49	844	560	605.25	646	691	799.75	848.3
	50–59	627	563.4	618	665	714	846.6	868.8
	60–69	289	555.5	614	662	714	839	895
	>70	56	544.55	598.75	677.5	729.75	831.65	859.45

Blood sugar (mg/dL)	20–29	796	60	70	77	84	95	98.09
	30–39	652	64	74	81	89	102	112
	40–49	844	67	78	85	94	116	137.95
	50–59	627	66	79	88	98	134.2	178.96
	60–69	289	65	78	89	99.5	146	155.9
	>70	56	69.7	79	86.5	94	130.65	143.77

SBP (mm/Hg)	20–29	796	100	110	120	130	148	152
	30–39	653	104	116	126	136	154	164
	40–49	844	110	120	130	142	169.75	176
	50–59	627	110	126	138	150	170	180
	60–69	289	110	126	140	150	179	182.6
	>70	56	109.1	128	140	150	164.7	180

DBP (mm/Hg)	20–29	796	68	70	80	80	90	96
	30–39	653	70	76	80	90	100	100
	40–49	844	70	80	84	90	100	102
	50–59	627	70	80	86	90	100	100.32
	60–69	289	70	80	84	90	100	105.8
	>70	56	67.4	80	82	90	100	100

BMI (kg/m^2^)	20–29	796	15.93	18.42	21.35	24.82	30.43	31.96
	30–39	653	17.45	21.35	23.93	26.65	32.11	33.92
	40–49	844	17.88	22.37	25	28.14	33.69	35.3
	50–59	627	19.22	22.78	25.51	28.22	33.33	34.66
	60–69	289	18.45	22.28	25.06	27.51	32.34	34.25
	>70	56	17.86	21.11	23.44	26.75	33.67	35.54

TC: Total Cholesterol; TG: Triglycerides; HDL-C: High Density Lipoprotein Cholesterol; LDL-C: Low Density Lipoprotein Cholesterol; VLDL: Very Low Density Lipoprotein; TL: Total Lipid; SBP: Systolic Blood Pressure; DBP: Diastolic Blood Pressure; BMI: Body Mass Index. Data are expressed as the 5th to 97th percentile of median.

**Table 6:** Comparison between mean lipid values of different studies.

	*N*	TC (mg/dL)	TG (mg/dL)	HDL-C (mg/dL)	LDL-C (mg/dL)	LDL/HDL	TC/HDL	VLDL (mg/dL)
Our study	3265	185.33 ± 39.245	115.6 ± 33.460	43.70 ± 9.870	118.50 ± 33.460	2.843 ± 1.028	4.429 ± 1.405	23.12 ± 12.70
Rajasthan	1527	168.13 ± 31.40	113 ± 47.44	45.10 ± 10.75	100.50 ± 27.71	—	—	—
Andhra Pradesh	1922	175.3 ± 34.6	132 ± 60.9	47.2 ± 10.4	101.1 ± 3.0	2.4 ± 1	4.15 ± 1.13	—
Bengal	1396	189.7 ± 33.3	132 ± 42.2	52.9 ± 10.1	115.6 ± 29.8	—	—	21.1 ± 6.5
Maharashtra	751	198 ± 37.16	119 ± 53.27	47 ± 11.13	121 ± 29.39	—	4.4 ± 1.13	—
Venezuela	434	191.96 ± 46	130.50 ± 102.53	44.27 ± 12.12	122.10 ± 38.40	—	—	26.10 ± 20.50
Nepal	454	184.0 ± 50.7	147.4 ± 88.7	45.0 ± 11.7	111.9 ± 42.0	—	—	—

TC: Total Cholesterol; TG: Triglycerides; HDL-C: High Density Lipoprotein Cholesterol; LDL-C: Low Density Lipoprotein Cholesterol; VLDL: Very Low Density Lipoprotein. Data are expressed as mean ± SD.

## References

[1] Reddy K. S., Yusuf S. (1998). Emerging epidemic of cardiovascular disease in developing countries. *Circulation*.

[2] North K. E., Howard B. V., Welty T. K. (2003). Genetic and environmental contributions to cardiovascular disease risk in American Indians the strong heart family study. *American Journal of Epidemiology*.

[3] Gupta R., Guptha S., Sharma K. K., Gupta A., Deedwania P. (2012). Regional variations in cardiovascular risk factors in India: India heart watch. *World Journal of Cardiology*.

[4] Zeljko H., Škarić-Jurić T., Narančić N. S. (2008). Traditional CVD risk factors and socio-economic deprivation in Roma minority population of Croatia. *Collegium Antropologicum*.

[5] Carmena R., Duriez P., Fruchart J.-C. (2004). Atherogenic lipoprotein particles in atherosclerosis. *Circulation*.

[6] Shai I., Rimm E. B., Hankinson S. E. (2004). Multivariate assessment of lipid parameters as predictors of coronary heart disease among postmenopausal women: potential implications for clinical guidelines. *Circulation*.

[7] Gupta R., Guptha S., Agrawal A., Kaul V., Gaur K., Gupta V. P. (2008). Secular trends in cholesterol lipoproteins and triglycerides and prevalence of dyslipidemias in an urban Indian population. *Lipids in Health and Disease*.

[8] Foulkes A. S., Wohl D. A., Frank I. (2006). Associations among race/ethnicity, ApoC-III genotypes, and lipids in HIV-1-infected individuals on antiretroviral therapy. *PLoS Medicine*.

[9] National Heart (2002). Third report of the National Cholesterol Education Program (NCEP) expert panel on detection, evaluation, and treatment of high blood cholesterol in adults (Adult Treatment Panel III) final report. *Circulation*.

[10] Yadav D., Gupta M., Mishra S., Sharma P. (2013). Reference interval for lipid profile in North Indian population from Rajasthan according to various partitioning criteria. *Clinica Chimica Acta*.

[11] Das M., Saikia M. (2009). Stimation of reference interval of lipid profile in Assamese population. *Indian Journal of Clinical Biochemistry*.

[12] Malati T., Mahesh M. R. U. (2009). Reference intervals for serum total cholesterol, HDL-cholesterol, LDL-cholesterol, triglycerides, Lp (a), apolipoprotein A-I, A-II, B, C-II, C-III, and E in healthy South Indians from Andhra Pradesh. *Indian Journal of Clinical Biochemistry*.

[13] de Maat M. P. M., Green F., de Knijff P., Jespersen J., Kluft C. (1997). Factor VII polymorphisms in populations with different risks of cardiovascular disease. *Arteriosclerosis, Thrombosis, and Vascular Biology*.

[14] Cruickshank J. K., Cooper J., Burnett M., Macduff J., Drubra U. (1991). Ethnic differences in fasting plasma C-peptide and insulin in relation to glucose tolerance and blood pressure. *The Lancet*.

[15] Joshi S. R., Saboo B., Vadivale M. (2012). Prevalence of diagnosed and undiagnosed diabetes and hypertension in India—results from the Screening India's Twin Epidemic (SITE) study. *Diabetes Technology and Therapeutics*.

[16] Bala D. V., Bodiwala I. L. A. N., Patel D. D., Shah P. M. (2006). Epidemiological determinants of tobacco use in Gujrat State, India. *Indian Journal of Community Medicine*.

[17] McKeigue P. M., Shah B., Marmot M. G. (1991). Relation of central obesity and insulin resistance with high diabetes prevalence and cardiovascular risk in South Asians. *The Lancet*.

[18] Jhala C. I., Shah U. V., Shah T. K., Naik B. K., Dafda J. D. (1998). A study of serum lipid profile part—1: establishment of normal reference values of serum lipid levels in healthy vegetarian population of Gujarat. *Indian Journal of Clinical Biochemistry*.

[19] Indian Consensus Group (1996). Indian consensus for prevention of coronary artery disease: a joint scientific statement of Indian society of hypertension and International College of Nutrition. *Journal of Nutritional and Environmental Medicine*.

[20] World Health Organization (2002). *The World Health Report: 2002: Reducing Risks, Promoting Healthy Life: Overview*.

[21] Kanjilal S., Shanker J., Rao V. S. (2008). Prevalence and component analysis of metabolic syndrome: an Indian atherosclerosis research study perspective. *Vascular Health and Risk Management*.

[22] Pandya H., Lakhani J. D., Patel N. (2011). Obesity is becoming synonym for diabetes in rural areas of india also an alarming situation. *International Journal of Biological and Medical Research*.

[23] Joshi A., Bhugra P., Lakhani J., Desai S. (2004). Body mass index and central obesity in hypertensive patients. *Gujarat Medical Journal*.

[24] Jhala C., Joshi P., Shah T., Naik B. (2009). Establishment of normal lipid reference values in healthy vegetarian population of rural western & northern Gujarat and comparison with available similar data of other parts of India. *Gujarat Medical Journal*.

[25] Kaur V., Verma M., Kaur A., Gupta S., Singh K. (2012). To establish the reference intervals of lipid profile in Punjab. *Indian Journal of Clinical Biochemistry*.

[26] Price J. F., Mowbray P. I., Lee A. J., Rumley A., Lowe G. D. O., Fowkes F. G. R. (1999). Relationship between smoking and cardiovascular risk factors in the development of peripheral arterial disease and coronary artery disease; Edinburgh Artery study. *European Heart Journal*.

[27] Betteridge D. (2004). The interplay of cardiovascular risk factors in the metabolic syndrome and type 2 diabetes. *European Heart Journal Supplements*.

[28] Sharma K. H., Sahoo S., Shah K. H. (2014). Are Gujarati Asian Indians ‘older’ for their ‘vascular age’ as compared to their ‘chronological age’?. *Quarterly Journal of Medicine*.

[29] Ashavaid T. F., Kondkar A. A., Todur S. P., Dherai A. J., Morey J., Raghavan R. (2005). Lipid, lipoprotein, apolipoprotein and lipoprotein (a) levels: reference intervals in a healthy Indian population. *Journal of Atherosclerosis and Thrombosis*.

[30] Goswami K., Bandyopadhyay A. (2003). Lipid profile in middle class Bengali population of Kolkata. *Indian Journal of Clinical Biochemistry*.

[31] Bermúdez V. J., Bello L. M., Naguib A. (2011). Lipid profile reference intervals in individuals from Maracaibo, Venezuela: an insight from the Maracaibo City Metabolic Syndrome prevalence study. *Revista Latinoamericana de Hipertension*.

[32] Limbu Y. R., Rai S. K., Ono K. (2008). Lipid profile of adult Nepalese population. *Nepal Medical College Journal*.

[33] Prajapati J., Dani S., Jain S. (2009). AS-209: risk factor profile for coronary artery disease in very young gujarati population. *The American Journal of Cardiology*.

